# Taxonomy and Phylogeny of Meruliaceae with Descriptions of Two New Species from China

**DOI:** 10.3390/jof8050501

**Published:** 2022-05-11

**Authors:** Zhan-Bo Liu, Jun-Li Zhang, Viktor Papp, Yu-Cheng Dai

**Affiliations:** 1 School of Ecology and Nature Conservation, Beijing Forestry University, Beijing 100083, China; zhanboliu@bjfu.edu.cn; 2 Institute of Vegetable Research, Jinzu Road 147, Lhasa 850000, China; 594686187@taaas.com; 3 Department of Botany, Hungarian University of Agriculture and Life Sciences, 1118 Budapest, Hungary; agaricum@gmail.com

**Keywords:** diversity, macrofungi, phylogenetic analyses, new taxa, wood-rotting fungi

## Abstract

Two new wood-inhabiting fungi *Hermanssonia fimbriata* sp. nov. and *Phlebia austroasiana* sp. nov. in the Meruliaceae family are described and illustrated from southwestern China based on molecular and morphological evidence. The characteristics of *H. fimbriata* include annual, resupinate basidiomata, the absence of cystidia and cystidioles, oblong ellipsoid basidiospores of 5–6 × 2.4–3 μm, and growth on rotten gymnosperm wood in the east Himalayas. Its basidiomata change drastically upon drying, from being a light-coloured, juicy, papillose-to-wrinkled hymenophore, to a dark-coloured, corky-to-gelatinous, and more or less smooth hymenophore. The characteristics of *Ph. austroasiana* include annual, resupinate basidiomata, a hydnoid hymenophore, 2–3 spines per mm, the presence of tubular cystidia of 20–25 × 3–3.5 µm, oblong ellipsoid basidiospores of 4.4–5.2 × 2.1–3 μm, and growth on angiosperm wood in tropical forests in the southern Yunnan Province. The phylogenetic analyses based on the combined 2-locus dataset (ITS1-5.8S-ITS2 (ITS) + nuclear large subunit RNA (nLSU)) confirm the placement of two new species, respectively, in *Hermanssonia* and *Phlebia* s. lato. Phylogenetically, the closely-related species to these two new species are discussed.

## 1. Introduction

The phlebioid clade within Polyporales includes three lineages at a family level, namely Phanerochaetaceae, Irpicaceae, and Meruliaceae [1,2]. The taxonomy of many of the genera belonging to these families is not currently settled, and a case in point example is the genus *Phlebia*. In a recent study, Chen et al. [3] concluded that *Phlebia* s.l. is still polyphyletic, with members addressed in all families of the phlebioid clade. Based on their multigene phylogenetic analysis, the core *Phlebia* clade belongs to the Meruliaceae with three additional clades: the *Hydnophlebia* clade, the *Mycoacia* clade, and the *Sarcodontia* clade. The core *Phlebia* clade included the genera *Aurantiopileus* Ginns et al., *Aurantiporus* Murrill, *Pappia* Zmitr., and *Phlebia* s.s., as well as some species of *Ceriporiopsis* Domański s.l. and *Mycoacia* s.l. [3].

*Phlebia* Fr. was erected by Fries [4] and typified by *Phlebia radiata* Fr. As the delimitation of the genus *Phlebia* s. str. is not yet clarified, in the present paper, we treat *Phlebia* sensu in the same way as Chen et al. [3]. The genus is characterized by white-rot, resupinate or rarely pileate basidiocarps with a tuberculate, merulioid, folded, odontioid or hydnoid hymenophore, a monomitic hyphal system, generative hyphae with clamp connections, neither amyloid nor dextrinoid, and allantoid to ellipsoid, hyaline, thin-walled, smooth, neither amyloid nor dextrinoid, acyanophilous basidiospores [3,5]. Formerly, several genera have been proposed to accommodate different lineages of *Phlebia* s. lato, but still many of the species has no modern interpretation, e.g., [3,6]. The monotypic genus *Hermanssonia* Zmitr. (Meruliaceae, Polyporales) was erected by Zmitrovich [7], based on *H. centrifuga* (P. Karst.) Zmitr. (=*Phlebia centrifuga* P. Karst.). The genus is characterized by white-rot, resupinate to effuse-reflexed, ceraceous to cartilaginous basidiomata, a phlebioid (radially-costate) or tuberculate hymenophore, a monomitic hyphal system, generative hyphae with clamp connections, and cylindrical, hyaline, thin-walled, smooth, neither amyloid nor dextrinoid basidiospores [7].

Four resupinate phlebioid specimens were collected from southwestern China (Tibet and Yunnan Province) during studies on wood-inhabiting fungi, and their morphology corresponded to concepts of *Hermanssonia* and *Phlebia*. Phylogenetic analyses based on the ITS1-5.8S-ITS2 (ITS) and nuclear large subunit RNA (nLSU) rDNA sequences were conducted to confirm their affinity. Both morphological and molecular evidence demonstrated that these four specimens represent two undescribed species of Meruliaceae. Thus, they are described in this paper.

## 2. Materials and Methods

### 2.1. Morphological Studies

Macro-morphological descriptions were based on voucher specimens and field notes. Microscopic structures were prepared from slide preparations of dried tissues stained with Cotton Blue and Melzer’s reagent as described by Wu et al. [8]. The following abbreviations are used in the description: CB = Cotton Blue; CB– = acyanophilous in Cotton Blue; IKI = Melzer’s reagent; IKI– = neither amyloid nor dextrinoid in Melzer’s reagent; KOH = 5% potassium hydroxide; L = mean spore length (arithmetic average of basidiospores); W = mean spore width (arithmetic average of basidiospores); and Q = variation in the L/W ratios between the specimens studied, (n = a/b) = number of spores (a) measured from given number of specimens (b). When the variation in spore size is shown, 5% of the measurements were excluded from each end of the range, and these values are shown in parentheses. Special colour terms follow Petersen [9] and herbarium abbreviations follow Thiers [10]. The voucher specimens for the present study are deposited in the herbarium of the Institute of Microbiology, Beijing Forestry University (BJFC), Beijing, China.

### 2.2. DNA Extraction, PCR, and Sequencing

Total genomic DNA was extracted from dried specimens using a CTAB Rapid Plant Genome Extraction Kit (Aidlab Biotechnologies Company, Ltd., Beijing, China) according to the manufacturer’s instructions with some modifications [11]. The ITS regions were amplified with primers ITS4 and ITS5 [12]. The nLSU regions were amplified with primers LR0R and LR7 [13].

The polymerase chain reaction (PCR) procedure for the ITS was as follows: initial denaturation at 95 °C for 3 min, followed by 35 cycles at 94 °C for 40 s, 54 °C for 45 s, 72 °C for 1 min, and a final extension of 72 °C for 10 min. The PCR procedure for the nLSU was as follows: initial denaturation at 94 °C for 1 min, followed by 35 cycles at 94 °C for 30 s, 48 °C for 1 min, and 72 °C for 1.5 min, and a final extension of 72 °C for 10 min [14]. The purification and sequencing of the PCR products was conducted by the Beijing Genomics Institute, Beijing, China, with the same primers used in the PCR reactions. Species were identified by sequence comparison with accessions in the NCBI databases using the BLAST program.

### 2.3. Phylogenetic Analyses

Phylogenetic trees were constructed using ITS + nLSU rDNA sequences, and phylogenetic analyses were performed with the Maximum Likelihood (ML), Maximum Parsimony (MP), and Bayesian Inference (BI) methods. Sequences of the species and strains were primarily adopted from ITS-based and 28S-based tree topology, as described by Huang et al. [5] and Chen et al. [3]. New sequences generated in this study, along with reference sequences retrieved from GenBank (Table 1), were aligned by MAFFT 7 (Katoh et al. [15]; http://mafft.cbrc.jp/alignment/server/, accessed on 18 April 2022) using the “G-INS-i” strategy and manually adjusted in BioEdit v. 7.2.5 [16]. Unreliably aligned sections were removed before the analyses, and efforts were made to manually inspect and improve the alignment. The data matrix was edited in Mesquite v3.70 (https://www.mesquiteproject.org/ (accessed on 18 April 2022). [17]. The sequence alignment was deposited at TreeBase. Sequences of *Hyphoderma mutatum* (Peck) Donk and *H. setigerum* (Fr.) Donk obtained from GenBank (https://www.ncbi.nlm.nih.gov/genbank/ (accessed on 18 April 2022) were used as outgroups to root the trees in the ITS + nLSU analysis.

**Table 1:** Taxa information and GenBank accession numbers of the sequences used in this study.

Species	Sample	GenBank Accession No.		References
		ITS	nLSU	
*Aurantiopileus mayaensi*	JV 1504/128	KT156706	—	—
*A. mayaensi*	TJB10228	HM772140	HM772139	[18]
*Aurantiporus croceus*	Miettinen-16483	KY948745	KY948901	[2]
*A. roseus*	Dai 13573	KJ698635	KJ698639	[19]
*Ceriporiopsis alboaurantia*	Cui 4136	KF845955	KF845948	[20]
*C. alboaurantia*	Cui 2877	KF845954	KF845947	[20]
*C. fimbriata*	Cui 1671	KJ698634	KJ698638	[19]
*C. fimbriata*	Dai 11672	KJ698633	KJ698637	[19]
*C. gilvescens*	BRNM 710166	FJ496684	FJ496684	[21]
*C. gilvescens*	BRNM 667882	FJ496685	FJ496719	[21]
*C. guidella*	HUBO 7659	FJ496687	FJ496722	[21]
*C. kunmingensis*	CLZhao 152	KX081072	KX081074	[22]
*C. kunmingensis*	CLZhao 153	KX081073	KX081075	[22]
*C. lagerheimii*	58240	KX008365	KX081077	[23]
*C. pseudoplacenta*	PRM 899297	JN592497	JN592504	[24]
*C. pseudoplacenta*	PRM 899300	JN592498	JN592505	[24]
*C. semisupina*	Cui 10222	KF845956	KF845949	[20]
*C. semisupina*	Cui 7971	KF845957	KF845950	[20]
*Climacodon septentrionalis*	AFTOL-767	AY854082	AY684165	[25]
*C. septentrionalis*	RLG-6890-Sp	KP135344	—	[26]
*Crustodontia chrysocreas*	HHB-3946	KP135357	—	[26]
*C. chrysocreas*	HHB-6333-Sp	KP135358	KP135263	[26]
*C. nigrodontea*	CLZhao 2758	MT896824	—	[5]
*C. nigrodontea*	CLZhao 2445	MT896821	MT896818	[27]
*C.* sp.	KUC20121123-24	KJ668482	—	[28]
*C. tongxiniana*	CLZhao 2255	MT020773	MT020751	[27]
*C. tongxiniana*	CLZhao 2316	MT020774	MT020752	[27]
*Geesterania carneola*	MCW 388/12	KY174999	KY174999	[29]
*G. davidii*	MCW 396/12	KY174998	KY174998	[29]
*Hermanssonia centrifuga*	CBS 125890	MH864088	MH875547	[30]
*H. centrifuga*	HHB-9239-Sp	KP135380	KP135262	[26]
***H. fimbriata***	**Dai 23266**	**ON135436**	**ON135440**	**Present study**
***H. fimbriata***	**Dai 23305**	**ON135437**	**ON135441**	**Present study**
***H. fimbriata***	**Dai 23306**	**ON135438**	**ON135442**	**Present study**
*Hydnophanerochaete odontoidea*	CLZhao 3882	MH784919	MH784929	[31]
*H. odontoidea*	CLZhao 4036	MH784927	MH784937	[31]
*Hydnophlebia chrysorhiza*	FD-282	KP135338	KP135217	[26]
*H. chrysorhiza*	HHB-18767	KP135337	—	[26]
*Hyphoderma mutatum*	HHB-15479-Sp	KP135296	KP135221	[26]
*H. setigerum*	FD-312	KP135297	KP135222	[26]
*Lilaceophlebia livida*	FCUG 2189	AF141624	AF141624	[21]
*L. livida*	FCUG 1290	HQ153414	—	[32]
*L. subserialis*	FCUG 1434	AF141631	AF141631	—
*Luteochaete subglobosa*	CLZhao 3639	MK881898	MK881788	[33]
*L. subglobosa*	CLZhao 3475	MK881897	MK881787	[33]
*Luteoporia albomarginata*	Dai 15229	KU598873	KU598878	[34]
*L. albomarginata*	GC 1702-1	LC379003	LC379155	[35]
*L. citriniporia*	Dai 19507	MT872218	MT872216	[36]
*L. citriniporia*	Dai 19622	MT872219	MT872217	[36]
*L. lutea*	CHWC 1506-68	MZ636997	MZ637157	[3]
*L. lutea*	GC 1409-1	MZ636998	MZ637158	[3]
*Mycoacia aurea*	DLL 2011263	KJ140747	—	[1]
*M. aurea*	RLG-5075-Sp	KY948759	MZ637161	[2,3]
*M. aurea*	DLL2011_100	KJ140614	—	[37]
*M. fuscoatra*	HHB 15354T	KP135367	—	[26]
*M.* cf. *kurilensis*	WEI 18-312	MZ637001	MZ637162	[3]
*M.* cf. *kurilensis*	WEI 18-324	MZ637002	MZ637163	[3]
*M. fuscoatra*	KHL 13275	JN649352	JN649352	[21]
*M. nothofagi*	HHB 12067	KP135370	—	[26]
*M. nothofagi*	KHL 13750	GU480000	GU480000	[21]
*Mycoaciella bispora*	EL13_99	—	AY586692	[38]
*M. efibulata*	WEI 19-057	MZ637012	MZ637172	[3]
*M. efibulata*	WEI 16-172	MZ637011	MZ637171	[3]
*Odoria alborubescens*	BP106943	MG097864	MG097867	[39]
*O. alborubescens*	BRNU 627479	JQ821319	JQ821318	[40]
*Pappia fissilis*	814	HQ728291	HQ729001	[41]
*P. fissilis*	BRNM 699803	HQ728292	HQ729002	[41]
*Phlebia acanthocystis*	KUC20131001-33	KJ668484	KJ668337	[26]
*P. acanthocystis*	FP150571	KY948767	KY948844	[2]
*P. acerina*	FD 301	KP135378	—	[2]
*P. acerina*	HHB 11146	KP135372	—	[26]
***P. austroasiana***	**Dai 17556**	**ON135439**	**ON135443**	**Present study**
***P. austroasiana***	E8898A	KJ654590	—	[42]
*P. brevispora*	HHB 7030	KP135387	—	[26]
*P. brevispora*	FBCC1463	LN611135	LN611135	[43]
*P. floridensis*	HHB 7175	KP135384	—	[26]
*P. floridensis*	HHB-9905-Sp	KP135383	KP135264	[26]
*P. fuscotuberculata*	CLZhao 10227	MT020759	MT020737	[27]
*P. fuscotuberculata*	CLZhao 10239	MT020760	MT020738	[27]
*P. hydnoidea*	HHB-1993-Sp	KY948778	KY948853	[2]
*P. lindtneri*	GB-1027	AB210076	—	[44]
*P. lindtneri*	GB-501	KY948772	KY948847	[2]
*P. ludoviciana*	HHB-8715-Sp	KY948770	KY948846	[2]
*P. ludoviciana*	FD-427	KP135342	—	[26]
*P. nantahaliensis*	HHB-2816-Sp	KY948777	KY948852	[2]
*P. radiata*	CBS 285.56	MH857642	MH869187	[30]
*P. radiata*	AFTOL-484	AY854087	AF287885	[25]
*P. radiata*	UBC: F19726	HQ604797	HQ604797	[1]
*P. rufa*	FBCC297	LN611092	LN611092	[43]
*P. rufa*	HHB-14924	KP135374	—	[26]
*P. serialis*	FCUG 2868	HQ153429	—	[32]
*P. serialis*	UC2023146	KP814195	—	[33]
*P. setulosa*	PH 11749	GU461312	—	[1]
*P. setulosa*	HHB-6891-Sp	KP135382	KP135267	[26]
*P. setulosa*	AH31879	GQ259417	GQ259417	[45]
*P. subochracea* I	KGN 162/95	EU118656	EU118656	[46]
*P. subochracea* II	FBCC295	LN611116	LN611116	[43]
*P. subochracea* II	HHB-8494-Sp	KY948768	KY948845	[2]
*P. tomentopileata*	CLZhao 9563	MT020765	MT020743	[27]
*P. tomentopileata*	CLZhao 9515	MT020764	MT020742	[27]
*P. tremellosa*	ES 20082	JX109859	JX109859	[1]
*P. tremellosa*	CBS 217.56	MH857589	MH869138	[30]
*Phlebiporia bubalina*	Dai 13168	KC782526	KC782528	[47]
*P. bubalina*	Dai 15179	KY131843	KY131902	[48]
*Sarcodontia uda*	FP-101544-Sp	KP135361	KP135232	[26]
*Sarcodontia uda*	USDA Kropp 1	KY948764	—	[2]
*Scopuloides hydnoides*	FP-150473	KP135355	KP135284	[26]
*S. hydnoides*	WEI 17-569	MZ637085	MZ637283	[3]
*Stereophlebia tuberculata*	FCUG 3157	HQ153427	—	[32]
*S. tuberculata*	Wu 1708-107	MZ637089	MZ637286	[3]

New sequences are in bold.

Maximum Parsimony analysis was applied to the ITS + nLSU dataset sequences. The approaches to phylogenetic analysis utilized those conducted by Chen and Cui [47], and the tree was constructed using PAUP* version 4.0 beta 10 [49]. All the characters were equally weighted, and gaps were treated as missing data. Trees were inferred using the heuristic search option with tree bisection and reconnection (TBR) branch swapping, and 1000 random sequence addition maxtrees were set to 5000. Branches of zero length were collapsed, and all the parsimonious trees were saved. Clade robustness was assessed using a bootstrap (BT) analysis with 1000 replicates [50]. Descriptive tree statistics, including the Consistency Index (CI), Homoplasy Index (HI), Rescaled Consistency index (RC), Retention Index (RI), and tree length (TL), were calculated for each Maximum Parsimonious Tree (MPT) generated.

The research using ML was conducted using RAxML-HPC v. 8.2.3 [51] and RAxML-HPC through the CIPRES Science Gateway ([52]; http://www.phylo.org, accessed on 18 April 2022). Statistical support values (BS) were obtained using nonparametric bootstrapping with 1000 replicates. The BI analysis was performed with MrBayes 3.2.7a [53]. Four Markov chains were run for two runs from random starting trees for 3 million generations until the split deviation frequency value < 0.01, and the trees were sampled at every 1000 generation. The first 25% of the sampled trees were discarded as burn-in, and the remaining ones were used to reconstruct a majority rule consensus tree and calculate the Bayesian Posterior Probabilities (BPP) of the clades. 

A total of 24 models of evolution were scored using PAUP* version 4.0 beta 10 [49]. Optimal substitution models for the combined dataset were then determined using the Akaike Information Criterion (AIC) implemented in MrModeltest 2.3 [54,55]. The model GTR + I + G was selected for use in the Maximum Likelihood (ML) and Bayesian Inference (BI) analyses.

Branches that received bootstrap support for Maximum Likelihood (BS), Maximum Parsimony (BP), and Bayesian Posterior Probabilities (BPP) > 75% (BS), 50% (BP), and 0.9 (BPP) were considered to be significantly supported. In addition, the ML analysis resulted in the best tree, and only the ML tree is shown along with the support values from the MP and BI analyses. FigTree v1.4.4 [56] was used to visualize the resulting tree.

## 3. Results

### 3.1. Phylogenetic Analyses

The combined ITS + nLSU dataset included sequences from 110 specimens representing 61 taxa (Table 1). The dataset had an aligned length of 2349 characters, of which 1503 were constant, 195 were variable but parsimony-uninformative, and 651 were parsimony-informative. MP analysis yielded nine equally parsimonious trees (TL = 3586, CI = 0.377, RI = 0.752, RC = 0.283, HI = 0.623). The best model for the ITS + nLSU dataset estimated and applied in the Bayesian analysis was GTR + I + G. Bayesian analysis and MP analysis resulted in a similar topology to the ML analysis, with an average standard deviation of split frequencies of 0.006112 (BI).

**Figure 1:**
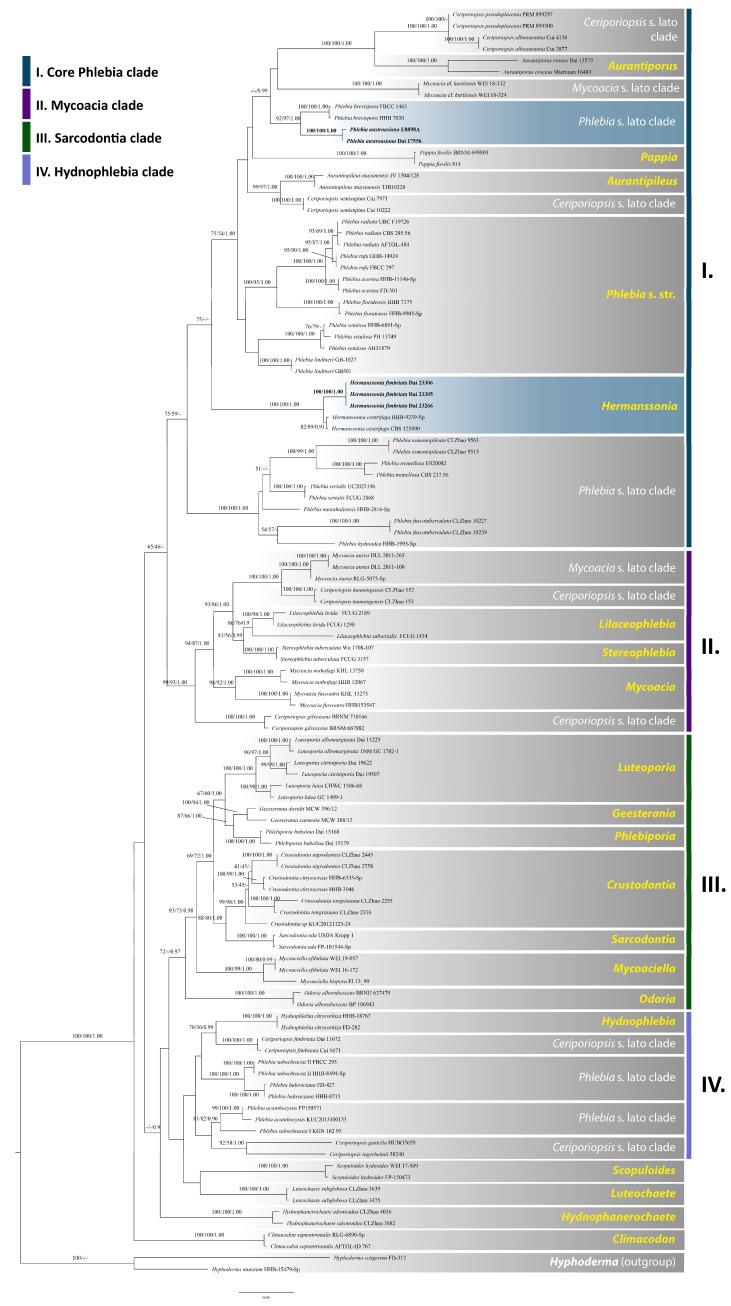
Phylogeny of Meruliaceae by MP analysis based on combined ITS and nLSU rDNA sequences. Branches are labelled with maximum likelihood bootstrap > 75%, parsimony bootstrap proportions > 50%, and Bayesian posterior probabilities > 0.9, respectively. New species are in bold.

The phylogeny (Figure 1) inferred from the ITS and nLSU sequences demonstrated that the new species, *Hermanssonia fimbriata* and *Phlebia austroasiana*, clustered into the genera *Hermanssonia* and *Phlebia*, respectively. *Hermanssonia fimbriata* grouped with *H. centrifuga* with strong support (100% BS, 100% BP, and 1.00 BPP, Figure 1) and *Phlebia austroasiana* grouped with *Ph. brevispora* Nakasone with strong support (92% BP, 97% BS, 1.00 BPP, Figure 1).

### 3.2. Taxonomy

**Figure 2:**
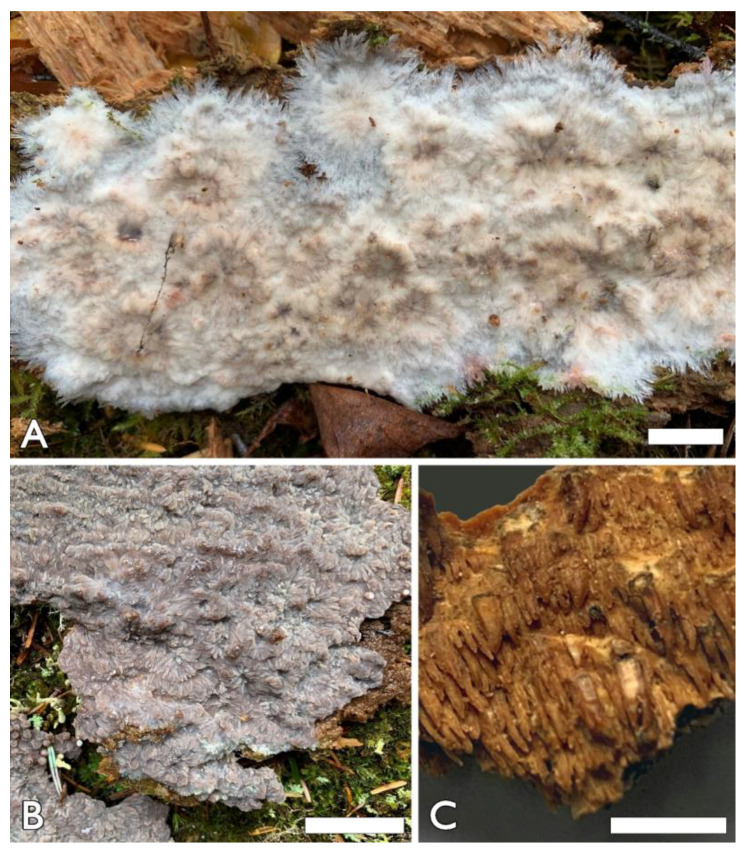
Basidiomata of *Hermanssonia fimbriata* and *Phlebia austroasiana*. (**A**) Juvenile basidiomata of *Hermanssonia fimbriata* (Holotype, Dai 23266). (**B**) Mature basidiomata of *H. fimbriata* (Paratype, Dai 23305). (**C**) Basidiomata of *Phlebia austroasiana* (Holotype, Dai 17556). Scale bars = 1.0 cm (**A**,**B**); 0.5 cm (**C**). Photo by: Yu-Cheng Dai (**A**,**B**) and Zhan-Bo Liu (**C**).

**Figure 3:**
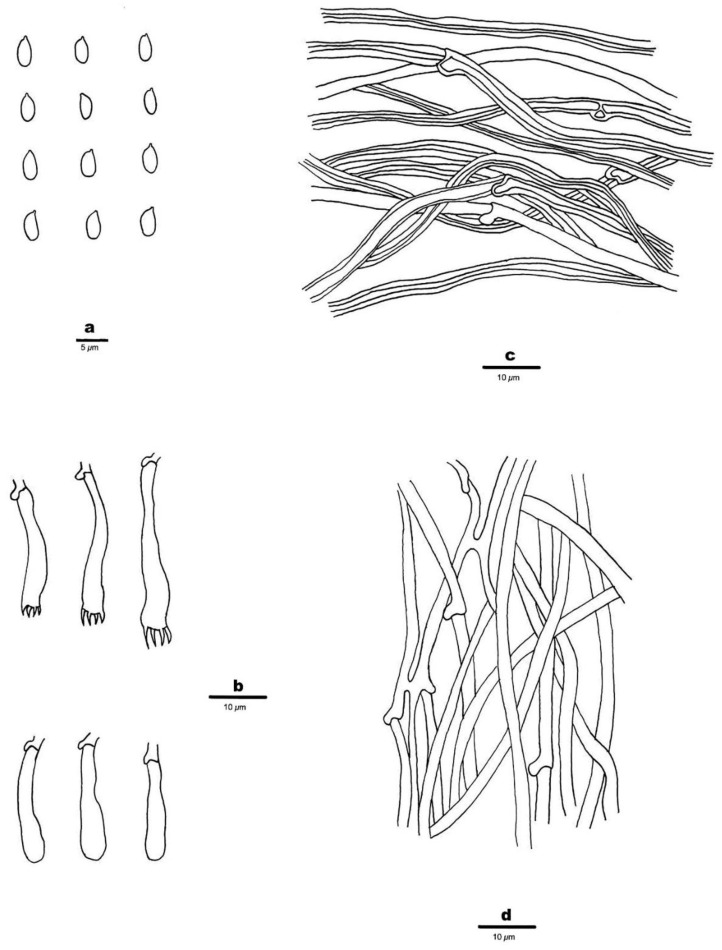
Microscopic structures of *Hermanssonia fimbriata* (Holotype, Dai 23266). (**a**) Basidiospores. (**b**) Basidia and basidioles. (**c**) Hyphae from subiculum. (**d**) Hyphae from subhymenium. Drawings by: Zhan-Bo Liu.

1. ***Hermanssoniafimbriata*** Z.B. Liu & Y.C. Dai, sp. Nov. (Figure 2A,B and Figure 3)

MycoBank number: MB 844038.

Diagnosis—*Hermanssonia fimbriata* is characterized by annual, resupinate basidiomata, a monomitic hyphal system with clamp connections, the absence of cystidia and cystidioles, and basidiospores which are oblong ellipsoid, hyaline, thin-walled, smooth, IKI–, CB–, and 5–6 × 2.4–3 μm. Its basidiomata change drastically upon drying, from being a light-coloured, juicy, papillose-to-wrinkled hymenophore, to a dark-coloured, corky-to-gelatinous, and more or less smooth hymenophore.

Etymology—*Fimbriata* (Lat.): refer to the species having fimbriate margin.

Type—China. Tibet, Linzhi, Milin County, Nanyi Valley, ca. 94°22′E, 29°37′N, elev. 3000 m, on rotten wood of *Picea*, 22 October 2021, Dai 23266 (BJFC 037837).

Basidiomata—Annual, resupinate, adnate, when fresh ceraceous and salmon (6A4) when juvenile, gelatinous, darkening to pale mouse grey (7C2) to light vinaceous grey (13B2/3) when mature, becoming corky, salmon (6A4) and reddish brown (8/9E7) upon drying, first as small colonies, later confluent up to 10 cm or more in the longest dimension, 4 cm in the widest dimension, and less than 0.1 mm thick at center when dry; hymenial surface irregularly papillose and partly radially or unevenly wrinkled; margin white and fimbriate; subiculum very thin to almost absent.

Hyphal structure—Hyphal system monomitic; generative hyphae with clamp con-nections, IKI–, CB–; tissue unchanged in KOH.

Subiculum—Generative hyphae hyaline, thin- to thick-walled, smooth, rarely branched, loosely interwoven, 2–4 μm in diam.

Hymenium—Generative hyphae in subhymenium hyaline, thin-walled, smooth, oc-casionally branched, loosely interwoven, 1.5–3 μm in diam; cystidia and cystidioles ab-sent; basidia clavate, hyaline, bearing four sterigmata and a basal clamp connection, 25–30 × 5–6 μm; basidioles in shape similar to basidia, but slightly shorter.

Basidiospores—Ellipsoid to oblong ellipsoid, hyaline, thin-walled, smooth, IKI–, CB–, (4.5–) 5–6 × (2.2–) 2.4–3 μm, L = 5.51 μm, W = 2.78 μm, Q = 1.88–2.04 (n = 60/2).

Additional specimens (paratypes) examined—China. Tibet, Linzhi, Milin County, Nanyi Valley, ca. 94°22′E, 29°37′N, elev. 3000 m, on rotten wood of *Picea*, 22 October 2021, Dai 23305 (BJFC 037876), Dai 23306 (BJFC 037877).

**Figure 4:**
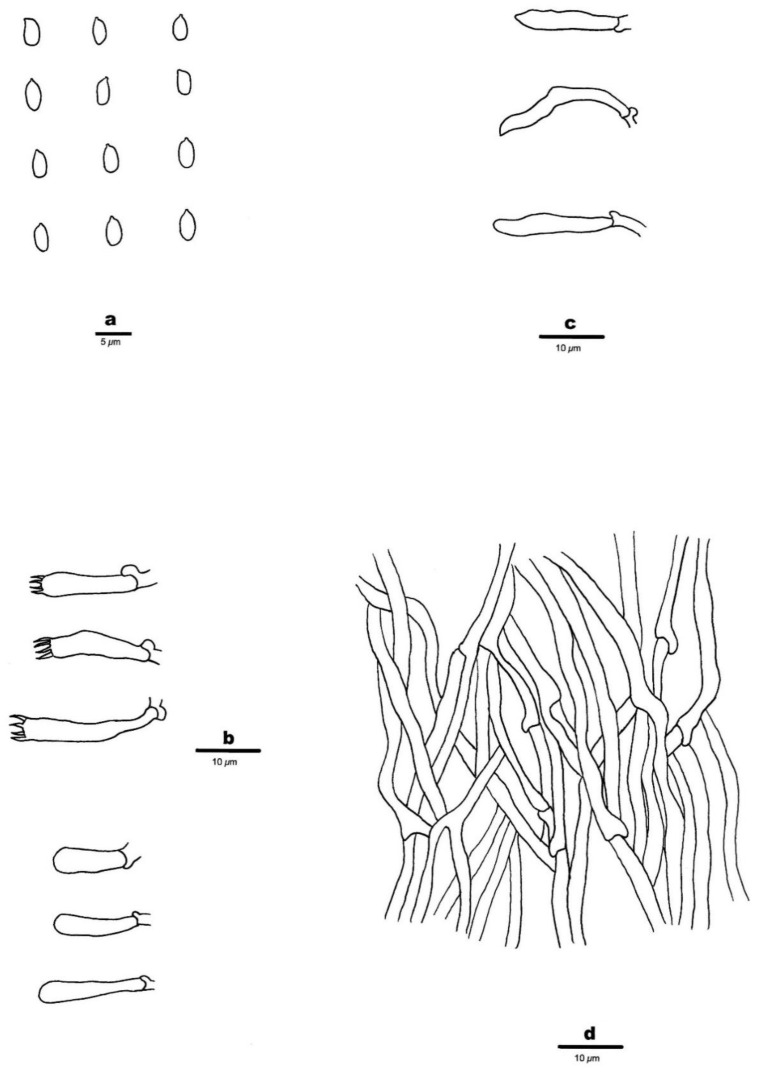
Microscopic structures of *Phlebia austroasiana* (Holotype, Dai 17556). (**a**) Basidiospores. (**b**) Basidia and basidioles. (**c**) Cystidia. (**d**) Hyphae from spine trama. Drawings by: Zhan-Bo Liu.

2. *Phlebia austroasiana* Z.B. Liu & Y.C. Dai, sp. Nov. Figure 2C and Figure 4

MycoBank number: MB 844039.

Diagnosis—*Phlebia austroasiana* is characterized by annual, resupinate basidiomata, a hymenophore with spines, 2–3 spines per mm, a monomitic hyphal system with clamp connections, the presence of tubular cystidia of 20–25 × 3–3.5 µm, and basidiospores which are oblong ellipsoid, hyaline, thin-walled, smooth, IKI–, CB–, 4.4–5.2 × 2.1–3 μm.

Etymology—*Austroasiana* (Lat.): refer to the species which is distributed in southeast Asia.

Type—China. Yunnan Province, Jinghong, Primeval Forest Park, ca. 100°52′E, 22°01′N, elev. 763 m, on angiosperm stump, 17 June 2017, Dai 17556 (BJFC 025088).

Basidiomata—Annual, resupinate, tightly adnate, gelatinous when dry, up to 5 cm long, 4 cm wide; hymenophore hydnoid, clay buff (6D4) when dry, not cracked; margin indistinct; spines crowded, clay buff (6D4), subulate, mostly separated, rarely fused, up to 2 mm long, 2–3 per mm at the base. Subiculum white, very thin to almost absent.

Hyphal structure—Hyphal system monomitic; generative hyphae with clamp con-nections, IKI–, CB–; tissue unchanged in KOH.Spines—Generative hyphae in spine trama hyaline, thin-walled, smooth, frequently branched, loosely interwoven, 2–3.5 μm in diam; cystidia tubular, thin-walled, with a basal clamp connection, 20–25 × 3–3.5 µm; cystidioles absent; basidia clavate, hyaline, bearing four sterigmata and a basal clamp connection, 18–26 × 4–5 μm; basidioles in shape similar to basidia, but slightly shorter.

Basidiospores—Ellipsoid to oblong ellipsoid, hyaline, thin-walled, smooth, IKI–, CB–, (4.1–)4.4–5.2 × (2–)2.1–3 μm, L = 4.86 μm, W = 2.53 μm, Q = 1.92 (n = 60/1).

## 4. Discussion

Chen et al. [3] divided the taxa of Meruliaceae into four clades: the core *Phlebia* clade, the *Hydnophlebia* clade, the *Mycoacia* clade, and the *Sarcodontia* clade. Two new species, *Hermanssonia fimbriata* and *Phlebia austroasiana*, are described in this study, based on morphological characters and phylogenetic analyses. Phylogenetically, they are nested in the core *Phlebia* clade, based on the ITS + nLSU sequence data (Figure 1). 

Phylogenetically, three specimens of *Hermanssonia fimbriata* formed a lineage with strong support (100% BS, 100% BP, and 1.00 BPP, Figure 1) and grouped with *H. centrifuga* with strong support (100% BS, 100% BP, and 1.00 BPP). Both species share annual, resupinate basidiomata, a monomitic hyphal system, generative hyphae with clamp connections, thin-walled, IKI–, CB– basidiospores, and growth on rotten gymnosperm wood [57]. *Hermanssonia fimbriata* can be distinguished from *H. centrifuga* by its shorter basidiospores (5–6 × 2.4–3 µm vs. 6.5–9 × 2.5–3 µm, [57]). *Hermanssonia centrifuga* was described as *Phlebia centrifuga* P. Karst. from Finland [58], and an Asian taxon, *Phlebia macra* Litsch., was described from Siberia [59]. The latter was treated as a synonym of *Ph. centrifuga* [60]. *Phlebia macra* differs from *Hermanssonia fimbriata* by larger basidiospores (6–7.5 × 3–3.2 µm vs. 5–6 × 2.4–3 µm, [59]). Morphologically, *H. fimbriata* is similar to *Phlebia coccineofulva* Schwein., *Ph. femsjoeensis* (Litsch. & S. Lundell) J. Erikss. & Hjortstam, and *Ph. radiata.* These four species share the phlebioid hymenophore, but the last three species have cystidia, while cystidia are absent in *Hermanssonia fimbriata.* Above all, basidiospores of *H. fimbriata* are larger than that of *Phlebia femsjoeensis* (4–5 × 2–2.5 µm, [61]) and *Ph. radiata* (4–5 × 1.8–2 µm, [61]), but thinner than that of *Ph. coccineofulva* (2.8–3.5 µm in width, [61]). *Hermanssonia fimbriata* also resembles *Phlebia subserialis* (Bourdot & Galzin) Donk and *Luteochaete subglobosa* (Sheng H. Wu) C.C. Chen & Sheng H. Wu (=*Phlebia wuliangshanensis* C.L. Zhao) by the resupinate and ceraceous basidiomata when fresh, a monomitic hyphal system, and generative hyphae with clamp connections; however, cystidia are abundant in *L. subglobosa* and *Phlebia subserialis*, while cystidia are absent in *Hermanssonia fimbriata.* In addition, basidiospores of *H. fimbriata* are wider than that of *Phlebia subserialis* (2.4–3 µm vs. 2–2.5 µm, [61]), but thinner than that of *Luteochaete subglobosa* (2.4–3 µm vs. 3–3.7 µm, [5]). *Hermanssonia* remained a monotypic genus until the present paper which contributes the second species in the genus.

An ITS sequence KJ654590 of sample E8898A, named *Phlebia* sp. from GenBank, is almost identical to Dai 17556 in the ITS regions and the similarity between them is up to 99.65%. Hence, we believe the sample E8898A collected from Indonesia [42] represents the same species as our specimen (Dai 17556) collected from the Yunnan Province, China. Both samples were collected in tropical Asia, and formed a lineage with strong support (100% BS, 100% BP, and 1.00 BPP, Figure 1) in our phylogeny. Hence, *Phlebia austroasiana* is described based on these two samples. *Ph. austroasiana* is closely related to *Ph. brevispora* (92% BP, 97% BS, 1.00 BPP, Figure 1), however, morphologically, *Ph. brevispora* differs from *Ph. austroasiana* by its tuberculate hymenophore [62], while *Ph. austroasiana* has a hydnoid hymenophore. In addition, *Ph. austroasiana* is distinguished from *Ph. brevispora* by its larger basidiospores (4.4–5.2 × 2.1–3 µm vs. 4–4.5 × 2–2.5 µm, [62]). Morphologically, *Ph. austroasiana* is similar to *Ph. capitata* Bernicchia & Gorjón. in macromorphology, but the cystidia in *Ph. capitata* are capitate [61], while the cystidia in *Ph. austroasiana* are tubular. In addition, *Ph. austroasiana* is distinguished from *Ph. capitata* by its smaller basidiospores (4.4–5.2 × 2.1–3 µm vs. 5–5.5 × 2.5–3 μm, [61]).

## Data Availability

Not applicable.
